# A preliminary prediction model of pediatric *Mycoplasma pneumoniae* pneumonia based on routine blood parameters by using machine learning method

**DOI:** 10.1186/s12879-024-09613-5

**Published:** 2024-07-18

**Authors:** Xuelian Peng, Yulong Liu, Bo Zhang, Chunyan Yang, Jian Dong, Chen Yong, Baoru Han, Jin Li

**Affiliations:** 1 Department of Laboratory Medicine, The Affiliated Dazu’s Hospital of Chongqing Medical University, Chongqing, 402360 China; 2 Medical Data Science Academy, College of Medical Informatics, Chongqing Medical University, Chongqing, 400016 China

**Keywords:** Routine blood parameters, Machine learning, Pediatric MPP infection, GBDT-based AI lab

## Abstract

**Background:**

The prevalence and severity of pediatric *Mycoplasma pneumoniae pneumonia* (MPP) poses a significant threat to the health and lives of children. In this study, we aim to systematically evaluate the value of routine blood parameters in predicting MPP and develop a robust and generalizable ensemble artificial intelligence (AI) model to assist in identifying patients with MPP.

**Methods:**

We collected 27 features, including routine blood parameters and hs-CRP levels, from patients admitted to The Affiliated Dazu’s Hospital of Chongqing Medical University with or without MPP between January, 2023 and January, 2024. A classification model was built using seven machine learning (ML) algorithms to develop an integrated prediction tool for diagnosing MPP. It was evaluated on both an internal validation set (982 individuals) and an external validation set (195 individuals). The primary outcome measured the accuracy of the model in predicting MPP.

**Results:**

The GBDT is state-of-the-art based on 27 features. Following inter-laboratory cohort testing, the GBDT demonstrated an AUC, accuracy, specificity, sensitivity, PPV, NPV, and F1-score of 0.980 (0.938–0.995), 0.928 (0.796–0.970), 0.929 (0.717-1.000), 0.926 (0.889–0.956), 0.922 (0.727-1.000), 0.937 (0.884–0.963), and 0.923 (0.800-0.966) in stratified 10-fold cross-validation. A GBDT-based AI Lab was developed to facilitate the healthcare providers in remote and impoverished areas.

**Conclusions:**

The GBDT-based AI Lab tool, with high sensitivity and specificity, could help discriminate between pediatric MPP infection and non-MPP infection based on routine blood parameters. Moreover, a user-friendly webpage tool for AI Lab could facilitate healthcare providers in remote and impoverished areas where advanced technologies are not accessible.

**Supplementary Information:**

The online version contains supplementary material available at 10.1186/s12879-024-09613-5.

## Background

*Mycoplasma pneumoniae* (MP) infection can cause upper and lower respiratory tract infections in children, and some cases can become severe. Early and accurate diagnosis of MP infection is of great significance to start rational antibiotic treatment as soon as possible. *Mycoplasma pneumoniae* pneumonia (MPP) is a common cause of community-acquired pneumonia in children, particularly among school-age children [1]. In recent years, MPP has emerged as a major type of community-acquired pneumonia (CAP) in East Asian children, with an increasing number of severe or refractory cases being reported, especially in China [2]. Notably, the significant surge of MP infections among Chinese children in 2023 has raised widespread concerns. Consequently, it is imperative for pediatricians to prioritize the clinical diagnosis of MPP. The diagnosis of MPP primarily relies on clinical and radiological manifestations, as well as microbiological and serological tests. However, there remains a lack of consensus on the definition and diagnosis of MPP. In recent years, many scholars have explored novel disease diagnosis models based on big data and machine learning algorithms in the medical field, achieving remarkable results in disease risk prediction and diagnosis [3]. Artificial intelligence (AI) utilizing machine learning (ML) encompasses techniques that autonomously learn patterns from data without requiring assumptions regarding data structure. One of the strengths of these techniques is their ability to capture nonlinear relationships and interactions among predictors. Numerous studies have demonstrated their promising performance in disease prediction [4–7]. In this study, we aimed to assess the effectiveness of routine blood parameters in the early identification of pediatric MPP infection using machine learning algorithms.

## Materials and methods

### Ethics approval

This study was carried out according to the protocol which was reviewed and approved by the Medical Ethics Committee of The Affiliated Dazu’s Hospital of Chongqing Medical University (Approval No. DZ2024-04-039). The Ethics Committee approved this study protocol and waived the obligation for informed consent because of the retrospective nature of the study.

### Predictive model construction process

This study employed machine learning techniques to develop a predictive model for childhood MP based on blood cell analysis parameters, aiming to provide intelligent support for the diagnosis of MPP. The overall flow chart of the predictive model is illustrated in Figure S1. The process involves constructing a dataset by collecting routine blood parameters and detecting MP RNA using RT-PCR. In the data preprocessing stage, the collected data undergoes preprocessing to prepare it for model training. Subsequently, multiple machine learning methods are utilized to construct the model, and the model with the best performance is selected. The specific training process of the model is detailed in Figure S2. Following model training, the trained model is exported to a deployable format, and an interface is developed for interaction with the deployed model. When inputting blood routine parameters into the deployed prediction model, accurate diagnoses of MPP and non-MPP cases can be obtained.

### Study population

We conducted a retrospective analysis of 27 features, including including routine blood parameters and hs-CRP levels, from patients admitted to The Affiliated Dazu’s Hospital of Chongqing Medical University with or without MPP between January, 2023 and January, 2024. At the same time, we examined the MP RNA results of throat swabs of all patients. All enrolled patients presented clinical symptoms suggestive of MP infection, such as fever and cough. The diagnostic criteria for MPP were based on positive MP RNA results from RT-PCR of pharyngeal swabs. Inclusion criteria for each group were as follows: (1) disease duration of < 6 days or > 10 days upon admission; (2) absence of chest imaging during days 6–10 of illness; (3) a medical history of asthma, tuberculosis, chronic malnutrition, aspiration, immunodeficiency, cystic fibrosis, primary ciliary dyskinesia, or bronchopulmonary dysplasia; (4) definite bacterial and/or adenovirus coinfection upon admission; and (5) loss to follow-up.

### Detection of routine blood parameters

Finger prick blood samples were collected for routine blood tests using a conventional analyzer (BC7500, MinDRay, China). The following parameters were measured: white blood cells (WBC), red blood cells (RBC), neutrophil ratio (NEUT%), lymphocyte ratio (Lymph%), monocyte ratio (Mono%), eosinophil ratio (Eos%), basophil ratio (Baso%), neutrophil absolute value (NEUT#), lymphocyte absolute value (Lymph#), monocyte absolute value (Mono#), eosinophil absolute value (Eos#), basophil absolute value (Baso#), nucleated erythrocyte absolute value (ARC), hemoglobin (Hb), hematocrit (HCT), mean corpuscular volume (MCV), mean corpuscular hemoglobin (MCH), mean corpuscular hemoglobin concentration (MCHC), red cell distribution width (RDW-CV), platelet count (PLT), plateletcrit (PCT), platelet-large cell ratio (P-LCR), platelet distribution width (PDW), mean platelet volume (MPV), and hypersensitive C-reactive protein (hs-CRP). Additionally, the following hematological parameters were calculated: lymphocyte-to-platelet ratio (LMR), neutrophil-to-lymphocyte ratio (NLR), Platelet-to-Lymphocyte Ratio (PLR), and monocyte-to-lymphocyte ratio (MLR).

### Detection of MP RNA using RT-PCR

A fully automatic nucleic acid extractor and its associated reagents (Shanghai ZJ Bio-Tech Co., Ltd.) were utilized to extract all nucleic acids from pharyngeal swabs. Throat swab specimens collected for MP nucleic acid determination underwent RT-PCR, employing MP nucleic acid detection kits (Guangzhou Da An Bio-Tech Co., Ltd.). The amplification system consisted of a final volume of 50 µl, comprising 27 µl of a mixture of influenza A and B virus nucleic acid fluorescent probes, 3 µl of the enzyme, and 20 µl of the sample. The amplification conditions were as follows: reverse transcription at 50 °C for 10 min, predenaturation at 95 °C for 15 min, denaturation at 95 °C for 15 s, and annealing, elongation, and fluorescence detection at 60 °C for 60 s, repeated for 40 cycles. All amplification reactions were conducted using a Gentier 96E quantitative PCR instrument (Xi’an TIANLONG Bio-Tech Co., Ltd.).

### Data preprocessing

The study included 982 patients (534 boys, 448 girls). Each participant in the raw dataset had 27 features and one label column. The label values were encoded as 1 for the positive class and 0 for the negative class. Python 3.11 and the open-source Python machine learning library, scikit-learn 1.4.0, were used for data preprocessing, modeling, evaluation, statistical analysis, and feature analysis. Subsequently, the data is processed using the RobustScaler data normalization method. This method removes the scale and bias from the data and reduces the distance between features by comparing the difference in scale between features.

### Machine learning algorithms

In terms of algorithms, initially, models were constructed using seven common machine learning algorithms separately: K-Nearest Neighbors (KNN), Naive Bayes (NB), Support Vector Machine (SVM), Decision Tree (DTM), Random Forest (RF), Xtreme Gradient Boosting (XGBoost), and Gradient Boosting Decision Tree (GBDT) [8]. Subsequently, various generic metrics were used to evaluate the performance of each ML algorithm on stratified 10-fold cross-validation. These metrics include the area under the receiver operating characteristic curve (AUC), accuracy, specificity, sensitivity, PPV, NPV and F1 score. AUC is a performance metric used to evaluate the quality of a learner, with higher AUC values indicating better classification performance of the model [9]. The probability threshold was set to 0.5. Accuracy represents the proportion of correctly identified samples among all samples and is one of the most common model evaluation metrics. Specificity evaluates the percentage of true negative patients correctly identified as negative. The higher the specificity, the lower the rate of misdiagnosis. Sensitivity measures the percentage of true positive patients correctly identified as positive. The higher the sensitivity, the lower the rate of underdiagnosis. PPV stands for positive predictive value, which represents the proportion of all positive patients correctly identified as positive. NPV stands for negative predictive value, which indicates the proportion of all patients identified as negative who are correctly predicted to be negative. The F1 score, a weighted average of precision and recall, combines the results of these two indicators.

### The training process of the model

The training process of the model, depicted in Figure S2, was divided into four stages: dataset construction, data processing, training and validation, and prediction. Hyperparameter tuning was conducted using random grid search, with the highest AUC value as the target. Seven metrics were derived from each hyperparameter-tuned machine learning model on the testing datasets. The average AUC value was calculated from ten repetitions of the model training process, and other evaluation metrics were similarly obtained.

The preprocessed data underwent model training and optimization using stratified 10-fold cross validation. The selected hyperparameters were assessed by comparing the trained model with the hyperparameter-tuned model. In the dataset, stratified 10-fold cross-validation involved randomly dividing all 982 subjects into 10 equally sized subsets. Firstly, nine of them were sequentially utilized as the training set, and one as the test set. This process was repeated 10 times, with each partition serving as the test set once. The average predictive performance over the 10 validation steps was considered the predictive performance of a classification algorithm. More reliable estimates of model performance were obtained through stratified 10-fold cross-validation in each repetition, ensuring consistent class distribution in each fold [10]. We evaluated the diagnostic ability of each model based on several indexes: AUC, accuracy, specificity, sensitivity, PPV, NPV, F1 score.

### Development of the GBDT-based AI lab

A novel ML-based algorithm, GBDT-based AI Lab, was developed using clinical information and routine blood parameters to assist in identifying patients with MPP.

The trained model is deployed to a server, and an AI Lab system is developed in a front-end and back-end separation form using Vue and Spring Boot. Through the visual interface, users can easily review, process, and analyze past medical cases. The system interfaces with the hospital Health Information System (HIS) to directly access laboratory data and facilitates the secure upload of pre-audited test data.

### Validation of the GBDT-based AI lab

The GBDT-based AI Lab was tested in an interlaboratory cohort in collaboration with The Affiliated Dazu’s Hospital of Chongqing Medical University to ensure reliability, reproducibility, and robustness.

## Results

### Patient characteristics

The diagnosis of pediatric MPP infection relies on the presence of MPP-like symptoms along with a positive RT-PCR result for MP. In this study, 448 children with MPP infection and 534 children without MPP infection were included. There were no significant differences in the age and sex distributions between the two clinical groups (Table 1). All 27 variables can be measured with routine blood tests in the pediatric department. Table 1 presents patients’ characteristics, including age, gender, and the 27 selected variables. The WBC, NEUT%, Lymph%, Mono%, Eos%, Baso%, NEUT#, Lymph#, NLR, PLR, MLR, Moni#, Eos#, Baso#, RBC, Hb, HCT, MCV, MCHC, PLT, PCT, P-LCR and hs-CRP level in the two clinical groups were significantly different, while the MCH, RDW-CV, MPV and PDW were not (Table 1).

**Table 1:** Distribution of patients’demographics characteristics and routine laboratory parameters

	Variables	Total	non-MPP group	MPP group	*P* value
	NO. of patients (%)	982 (100)	534 (54.379)	448 (45.621)	
1	Age, median, year	5.556 (3.176)	5.356 (3.580)	5.793 (2.594)	0.032
2	Gender, NO. (%)				
	Male	534 (100)	279 (52.247)	255 (47.753)	
	Female	448 (100)	255 (56.920)	193 (43.080)	
3	WBC, mean (SD),	7.976 (2.833)	7.381 (1.803)	8.686 (3.577)	< 0.001^*^
4	Neut%, mean (SD), %	0.490 (0.156)	41.014 (12.262)	58.567 (13.611)	< 0.001^*^
5	Lymph%, mean (SD), %	0.415 (0.152)	49.552 (12.219)	31.954 (12.546)	< 0.001^*^
6	Mono%, mean (SD), %	0.067 (0.024)	5.787 (1.354)	7.710 (2.865)	< 0.001^*^
7	Eos%, mean (SD), %	0.026 (0.022)	3.337 (2.281)	1.668 (1.803)	< 0.001^*^
8	NRBC%, mean (SD), %	0.000 (0.000)	0.000 (0.000)	0.003 (0.043)	0.094
9	Baso%, mean (SD), %	0.002 (0.002)	0.311 (0.240)	0.100 (0.116)	< 0.001^*^
10	Neut#, mean (SD), 10 ^9^/L	4.039 (2.467)	3.016 (1.204)	5.259 (2.980)	< 0.001^*^
11	Lymph#, mean (SD), 10 ^9^/L	3.200 (1.420)	3.673 (1.376)	2.635 (1.256)	< 0.001^*^
12	Mono#, mean (SD), 10 ^9^/L	0.527 (0.260)	0.425 (0.139)	0.648 (0.313)	< 0.001^*^
13	Eos#, mean (SD), 10 ^9^/L	0.195 (0.171)	0.244 (0.172)	0.137 (0.150)	< 0.001^*^
14	Baso#, mean (SD), 10 ^9^/L	0.016 (0.016)	0.022 (0.017)	0.008 (0.009)	< 0.001^*^
15	NLR, mean (SD)	1.611 (1.455)	0.958 (0.583)	2.388 (1.767)	< 0.001^*^
16	MLR, mean (SD)	0.197 (0.148)	0.126 (0.053)	0.282 (0.178)	< 0.001^*^
17	PLR, mean (SD)	108.930 (52.130)	94.453 (35.929)	126.186 (62.213)	< 0.001^*^
18	NRBC, mean (SD), 10^12^/L	0.000 (0.002)	0.000 (0.000)	0.000 (0.004)	0.060
19	RBC, mean (SD), 10^12^/L	4.845 (0.406)	4.768 (0.345)	4.937 (0.453)	< 0.001^*^
20	Hb, mean (SD), g/L	132.740 (9.886)	130.916 (9.225)	134.915 (10.203)	< 0.001^*^
21	HCT, mean (SD), %	40.380 (3.031)	39.498 (2.692)	41.431 (3.076)	< 0.001^*^
22	MCV, mean (SD), fl.	83.545 (4.934)	82.977 (4.350)	84.223 (5.474)	< 0.001^*^
23	MCH, mean (SD), pg	27.482 (1.892)	27.512 (1.686)	27.447 (2.111)	0.594
24	MCHC, mean (SD), g/L	328.852 (8.681)	331.470 (7.573)	325.732 (8.883)	< 0.001^*^
25	RDW-CV, mean (SD), fl.	13.271 (0.829)	13.236 (0.765)	13.313 (0.898)	0.150
26	PLT, mean (SD), 10 ^9^/L	299.878 (84.176)	309.691 (67.145)	288.181 (99.528)	< 0.001^*^
27	PCT, mean (SD), %	0.278 (0.072)	0.290 (0.058)	0.264 (0.083)	< 0.001^*^
28	PDW, mean (SD)	16.038 (0.707)	15.949 (0.337)	16.145 (0.969)	< 0.001^*^
29	P-LCR, mean (SD), %	21.879 (6.245)	22.141 (6.033)	21.567 (6.475)	0.152
30	MPV, mean (SD), fl.	9.368 (0.911)	9.426 (0.874)	9.298 (0.948)	0.029
31	hs-CRP, mean (SD), mg/L	4.633 (8.683)	1.292 (1.069)	8.615 (11.608)	< 0.001^*^

Absolute numbers and percentages are used for categorical variables and mean and standard deviation are used for continuous variables

^*^shows the signifcant diferences between the non-MPP group and MPP group

### Performance of different models

**Fig. 1:**
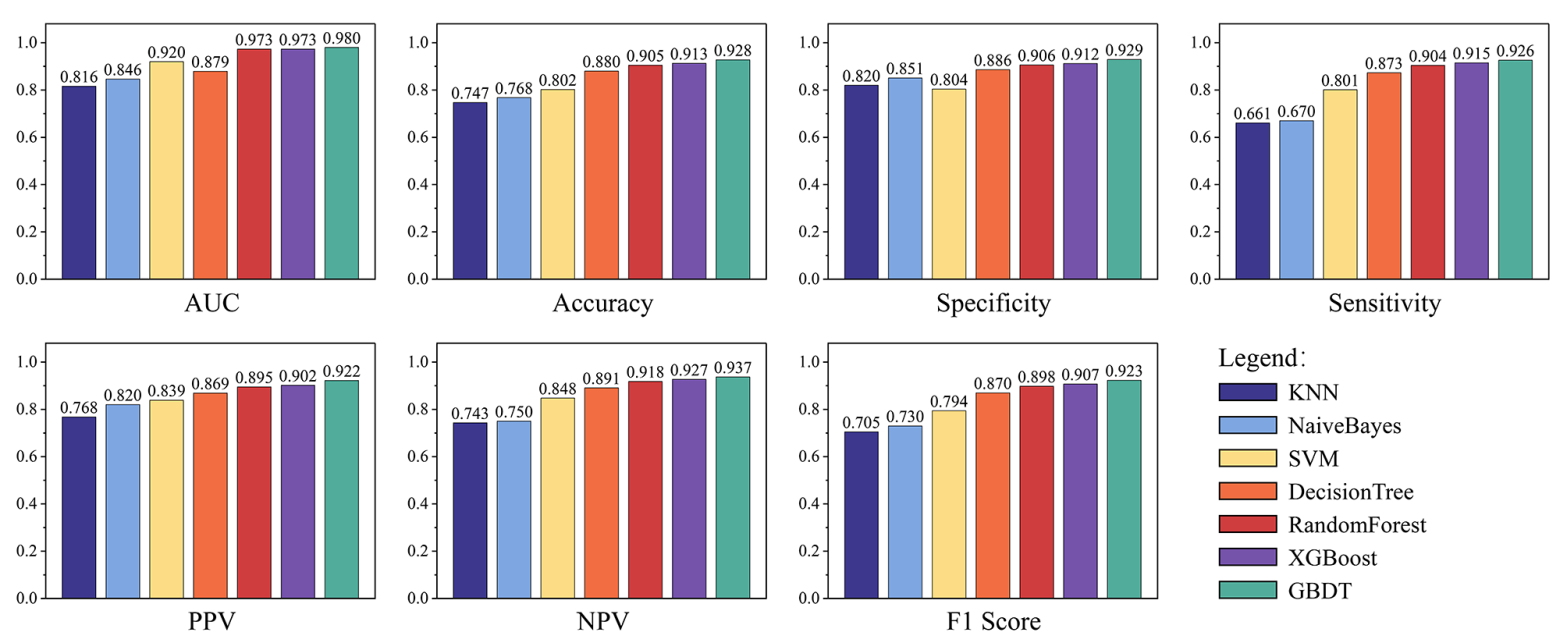
Performance of seven models. Through the assessment of the seven machine learning models using seven performance metrics, the diagnostic efficacy of the models can be evaluated across various dimensions, allowing for a comprehensive selection of the optimal approach. GBDT based on ensemble learning outperforms other methods across all metrics

**Fig. 2:**
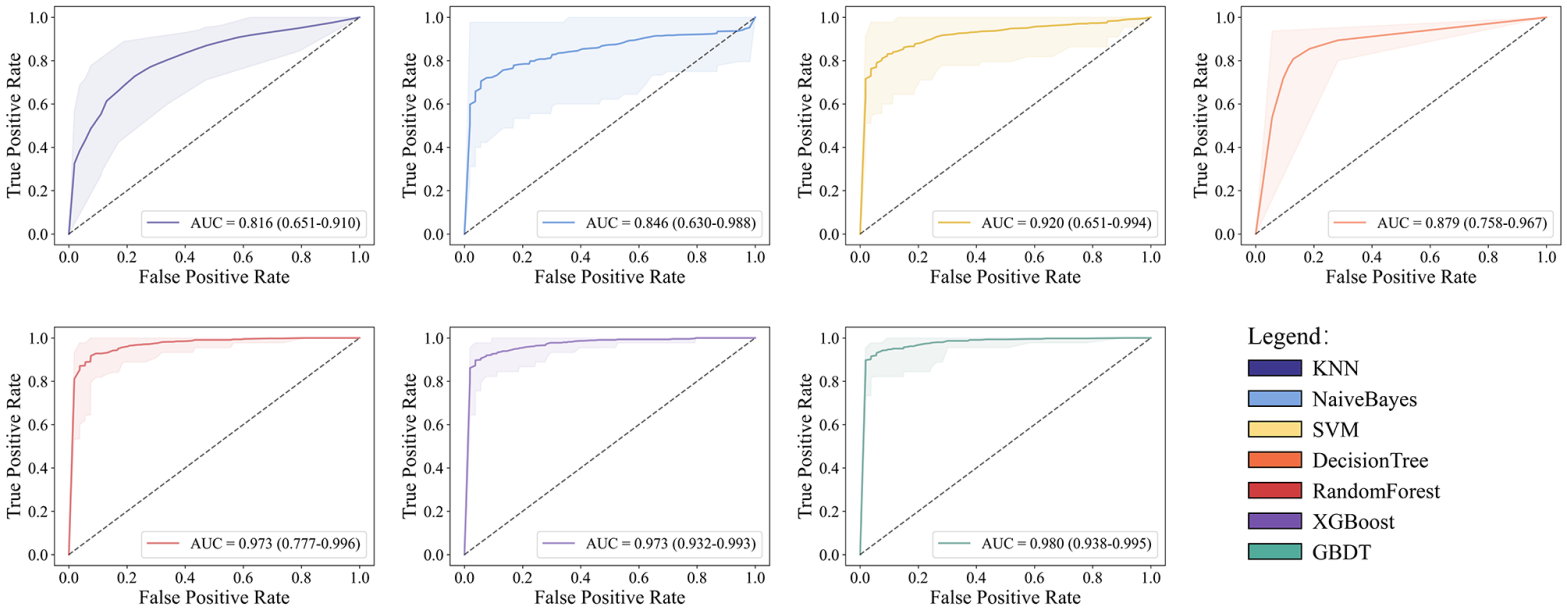
The average ROC curves for the seven models on 10-fold cross-validation are depicted. The shaded areas represent the range between the maximum and minimum values of the ROC curves during cross-validation. Comparative analysis reveals that the diagnostic performance of the integrated learning-based RandomForest, XGBoost, and GBDT models is more consistent compared to the single-base learner approach, with GBDT demonstrating superior AUC values

**Fig. 3:**
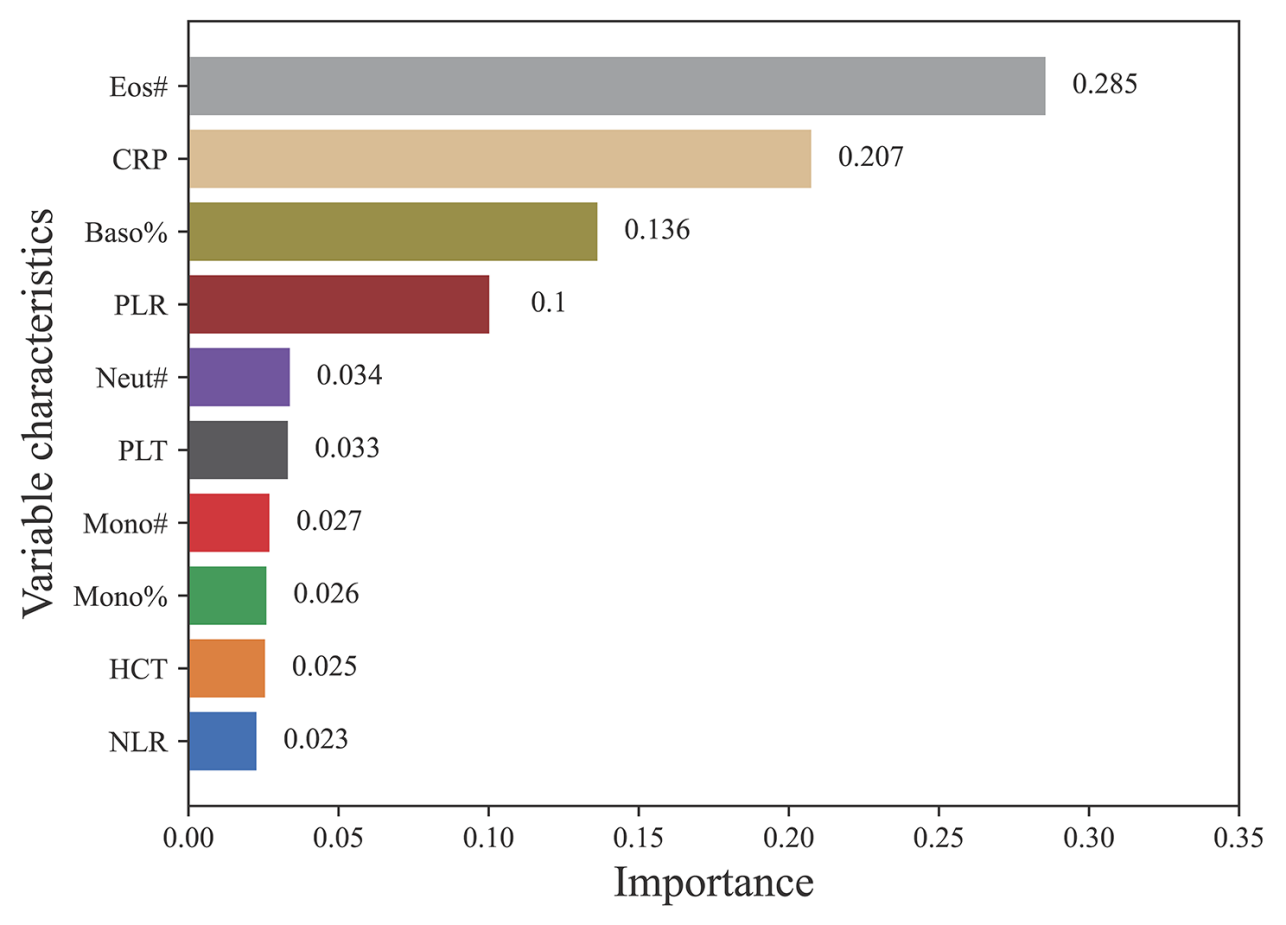
The top 10 variables in the decision-making process of GBDT-based models. Among them, Eos#, CRP, Baso%, and PLR stand out as the most important factors, playing a crucial role in model decisions

The developed machine learning models for classification and regression were evaluated with the following metrics: the area under the receiver operating characteristic curve (AUC), accuracy, specificity, sensitivity, PPV, NPV and F1 score. The average performance of stratified 10 fold cross validation for these models is shown in Table 2. The accuracy values of KNN, NB, SVM, DT, RF, XGBoost, and GBDT were 0.747, 0.768, 0.802, 0.880, 0.905, 0.913 and 0.928, respectively. All models achieved accuracy values equal to or greater than 0.740. The AUC values of KNN, NB, SVM, DT, RF, XGBoost, and GBDT were 0.816, 0.846, 0.920, 0.879, 0.973, 0.973 and 0.980, respectively. The GBDT model obtained the highest AUC (0.980), followed by RF and XGBoost with the second-best precision (0.973). These results indicated that GBDT was the best-performing model in the training set, achieving the highest AUC (0.980), accuracy (0.928), specificity (0.929), sensitivity (0.926), PPV (0.922), NPV (0.937) and F1 score (0.92) (Table 2). Moreover, other indices also demonstrated outstanding performance, indicating that the GBDT-based classification model exhibits high accuracy in distinguishing between MPP and non-MPP group. This model can be effectively utilized in constructing a diagnostic model for MPP. Figure 1 visualises the results of the evaluation metrics for all the compared models, allowing an intuitive comparison of the advantages of the best model. From Fig. 1, it is evident that the established GBDT model demonstrates exceptionally high performance across various indicators, rendering it suitable for constructing prediction models. To assess the performance of the various models, we plot the receiver operating characteristic (ROC) of all the compared models in Fig. 2 for performance comparison. In Fig. 2, the shaded areas with varying background colors represent the range of ROC curves in cross-validation. Comparison among the different models reveals that KNN, NB, SVM, and DT exhibit wider ranges of ROC errors, suggesting their classification performance is less stable and susceptible to data partitioning. On the other hand, RF, XGBoost, and GBDT, based on integrated learning principles, demonstrate relatively narrower error ranges, with GBDT showing the smallest errors. This indicates excellent classification performance, strong generalization, and higher practical value for these models. Furthermore, we displayed the feature weights of the optimal model output, arranging them in descending order to illustrate the contribution of different features to the model decision-making results. Figure 3 illustrates the top ten variables that contribute most significantly to this model, with Eos# and CRP making substantial contributions to discriminative diagnosis.

**Table 2:** Comparison of ten-fold cross-validation results of different machine algorithm models

	KNN	NaiveBayes	SVM	DecisionTree	RandomForest	XGBoost	GBDT
**AUC**	0.816 (0.651–0.910)	0.846 (0.630–0.988)	0.920 (0.651–0.994)	0.879 (0.758–0.967)	0.973 (0.777–0.996)	0.973 (0.932–0.993)	0.980 (0.938–0.995)
**Accuracy**	0.747 (0.633–0.857)	0.768 (0.684–0.888)	0.802 (0.602–0.960)	0.880 (0.755–0.970)	0.905 (0.684–0.960)	0.913 (0.806–0.969)	0.928 (0.796–0.970)
**Specificity**	0.820 (0.611–0.963)	0.851 (0.796-1.000)	0.804 (0.315-1.000)	0.886 (0.717-1.000)	0.906 (0.630–0.981)	0.912 (0.736-1.000)	0.929 (0.717-1.000)
**Sensitivity**	0.661 (0.489–0.778)	0.670 (0.533–0.818)	0.801 (0.489-1.000)	0.873 (0.800-0.933)	0.904 (0.750–0.956)	0.915 (0.867–0.956)	0.926 (0.889–0.956)
**PPV**	0.768 (0.600-0.939)	0.820 (0.706-1.000)	0.839 (0.538-1.000)	0.869 (0.706-1.000)	0.895 (0.623–0.977)	0.902 (0.741-1.000)	0.922 (0.727-1.000)
**NPV**	0.743 (0.660–0.831)	0.750 (0.672–0.843)	0.848 (0.660-1.000)	0.891 (0.890–0.947)	0.918 (0.756–0.963)	0.927 (0.886–0.962)	0.937 (0.884–0.963)
**F1 Score**	0.705 (0.587–0.833)	0.730 (0.608–0.864)	0.794 (0.587–0.957)	0.870 (0.750–0.966)	0.898 (0.680–0.956)	0.907 (0.808–0.966)	0.923 (0.800-0.966)

The ranges for mean, maximum, and minimum values from 10-fold cross-validation were calculated individually for various evaluation metrics

AUC: area under the curve; PPV: positive predictive value; NPV: negative predictive value;

KNN: K-Nearest Neighbor; SVM: Support Vector Machine Learning;

### A webpage tool of the GBDT-based AI lab

**Fig. 4:**
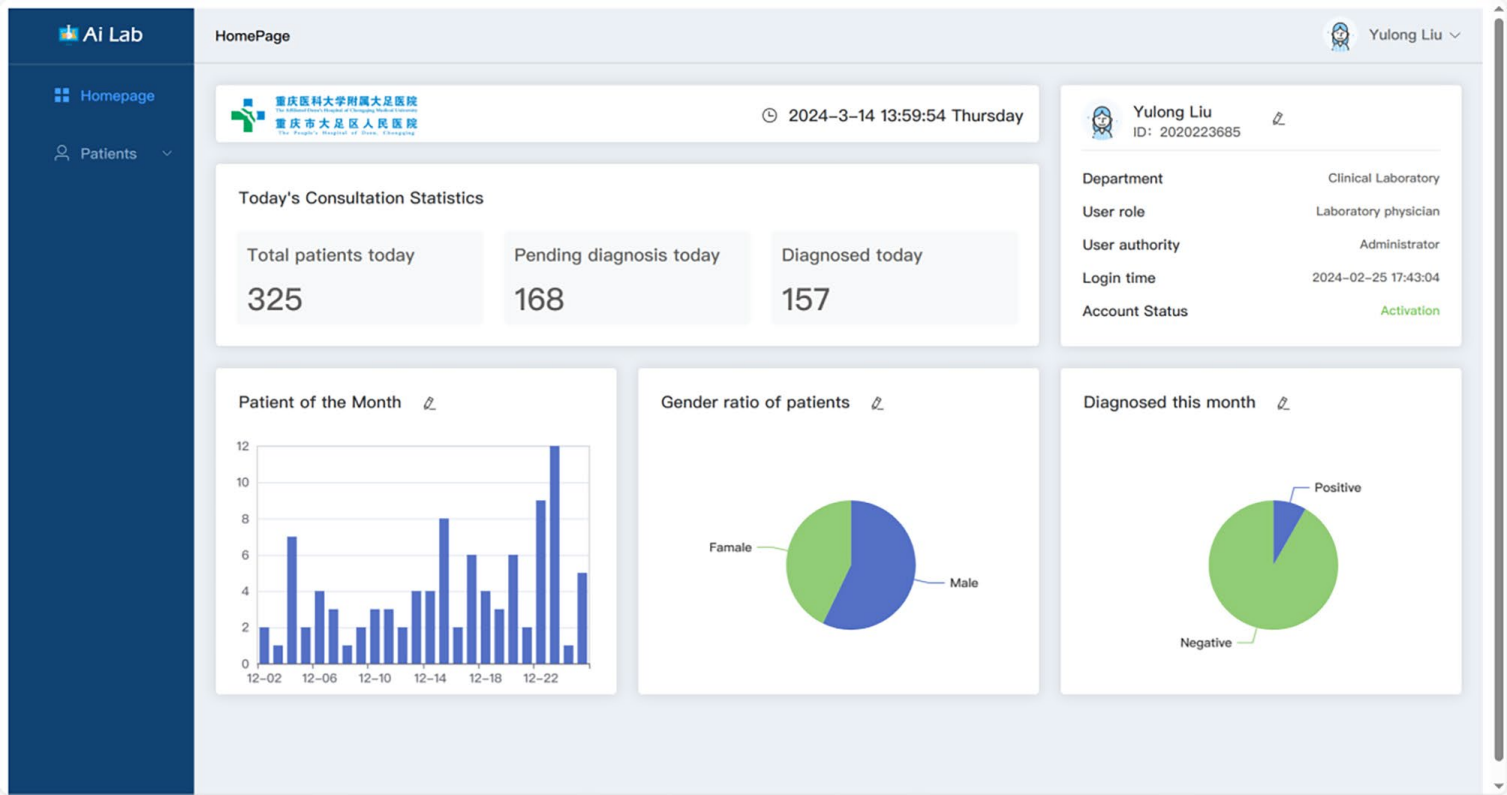
The main system interface provides a quick view of today’s patient statistics. It supports modifications to the dimensions and objects of the statistics for utilization on a large data screen

A webpage tool of the GBDT-based AI Lab was established to facilitate the healthcare providers in rural areas. A screenshot of the webpage was shown in Fig. 4. After inputting the necessary parameters, the patient could be discriminated as MPP or non-MPP with probability.

### Model deployment

**Fig. 5:**
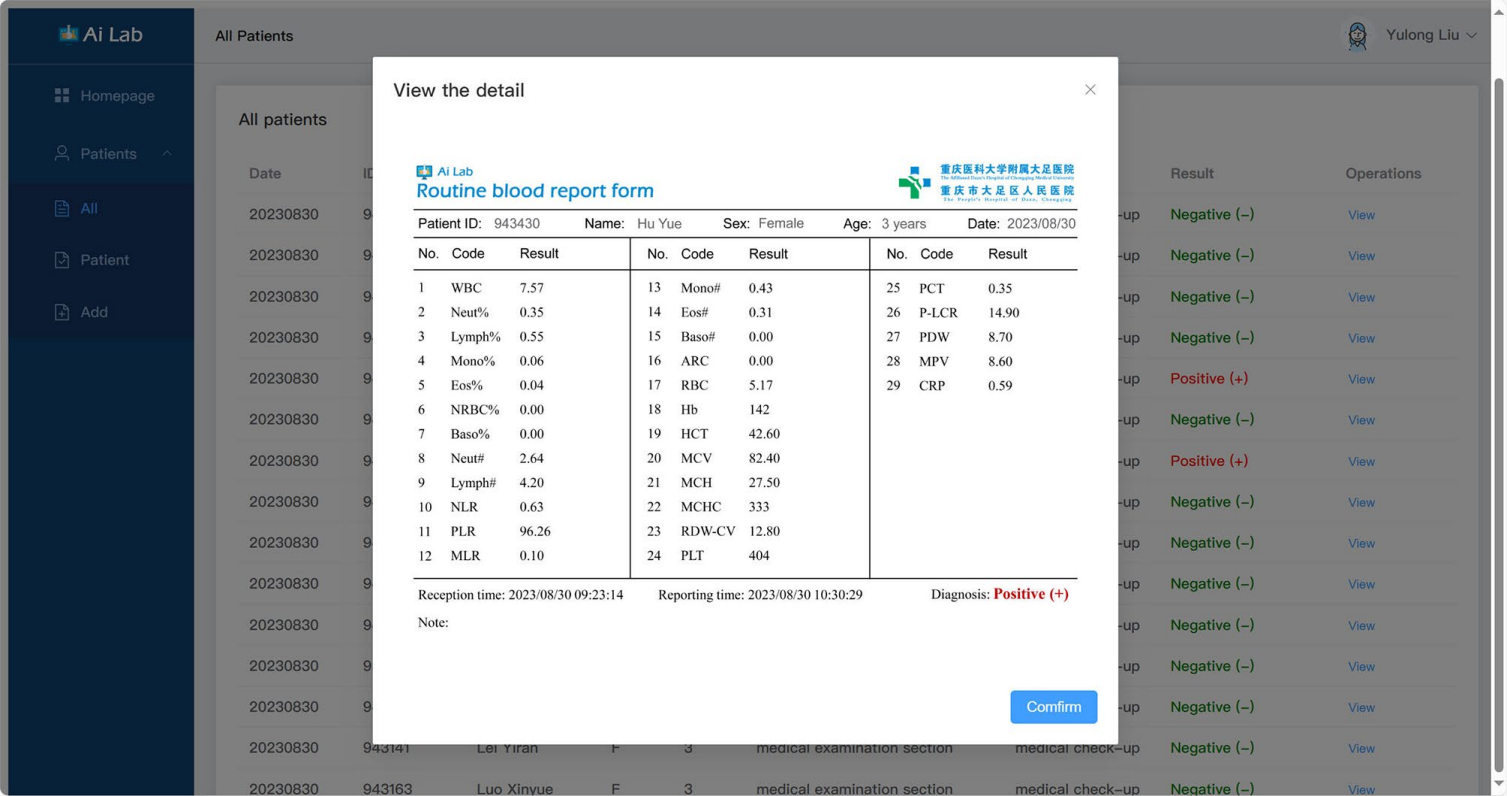
A detailed report of the patient’s findings is generated. Following the automatic diagnosis by the system, a specific diagnostic report is produced, including the patient’s personal details and the values of all parameters used for the diagnosis, aiding in documenting the disease progression

Users authorized by the administrator can access the system through the login screen. Users who forget their passwords or whose accounts are frozen due to risky operations can reset their account status through authentication. Upon successful login, users are directed to the main interface, displaying account data, current time, today’s consultation status, and a statistics interface in Fig. 4. The consultation status visually presents the total number of consultations, the number of people consulted, and the number of people waiting to be consulted on the same day. The statistics interface reads past cases from the database, allowing users to customize statistics dimensions and forms by clicking the edit icon. In this interface, users can quickly view past cases in the database. The statistics table is arranged by date by default, and only the patient’s personal information is previewed on the page. To view in detail, users can click the view button to access the detailed interface in Fig. 5. On the detail page, all test data will be displayed comprehensively. The system usually automatically updates patient data from the hospital database and performs model inference after the patient’s test is completed. If manual uploading of patient data is required, it can be done in the import patient interface. Patients can be imported by uploading a security-cleared CSV file for automatic decoding or by manually entering test results. After uploading, users can click the confirm button to update the data into the database and perform model inference. They can then return to the all patients interface to find the patients when the inference is complete.

### Interlaboratory validation

A webpage tool of the GBDT-based AI Lab was tested in an interlaboratory cohort with the The Affiliated Dazu’s Hospital of Chongqing Medical University. In evaluating the overall performance of GBDT-based AI Lab, predictive performances of the classification models on the internal validation set are summarised. 194 individuals as a publicly available MPP prediction tool. The GBDT-based AI Lab model showed high prediction efficacy in distinguishing individuals with MPP versus those who did not have MPP, obtaining an AUC of 0.980 for the internal validation set. Table 3 presents a comparison of GBDT performance between the internal validation dataset and the external validation dataset. The achieved AUC surpasses 0.95, indicating the robust generalization capability of the GBDT model when confronted with real-world data. Meanwhile, GBDT-based AI Lab also demonstrated superior and more stable performance than the seven competing methods, especially in AUC and sensitivity (Table 2). The GBDT-based AI Lab yielded a sensitivity of 0.926 and specificity of 0.929, with an AUC of 0.980 (Table 2).

**Table 3:** Comparison of results validated on internal and external validation datasets

	Internal validation (54 vs. 45)	External validation (137 vs. 58)
**AUC**	0.980	0.957
**Accuracy**	0.928	0.935
**Specificity**	0.929	0.950
**Sensitivity**	0.926	0.900
**PPV**	0.922	0.885
**NPV**	0.937	0.957
**F1 Score**	0.923	0.893

The internal validation set comprised 54 non-MPP patients and 45 MPP patients. The external validation set included 137 non-MPP patients and 58 MPP patients

AUC: area under the curve; PPV: positive predictive value; NPV: negative predictive value

## Discussion

MP is a pathogen exhibiting characteristics bridging those of bacteria and viruses. It demonstrates resilience, capable of surviving and replicating DNA in vitro under minimal environmental conditions [11, 12]. Moreover, MP shares antigenic similarities with certain human tissues, leading to the production of antibodies that can impair the human immune response [13]. Additionally, MP can trigger glucose fermentation, resulting in the generation of peroxidase and lactic acid, contributing to tissue and organ damage, multi-organ involvement, or respiratory distress, particularly affecting children’s health [14]. Therefore, early diagnosis and treatment of MPP are essential. In clinical practice, various methods are employed for diagnosing MPP, including MP culture, serological antibody detection, and nucleic acid detection [15–17]. While PCR detection offers high sensitivity, its reliance on specific environmental conditions poses limitations. Additionally, MP culture, considered the “gold standard” for diagnosis, has a lengthy detection cycle that may delay treatment initiation. Serological antibody detection, while practical, requires detection several days after infection. This study proposes a predictive model for diagnosing pediatric MPP using different machine learning algorithms. The results indicate that the GBDT model has a better predictive effect than the KNN, NB, SVM, DT, RF and XGBoost. Currently, research on MPP prediction mainly focuses on regression methods. Logistic regression, a classical linear regression algorithm commonly used in MPP prediction research, struggles to address data imbalance effectively. GBDT model is an iterative decision tree algorithm that can achieve good results on many data by combining gradient boosting and the advantages of decision trees. They are also versatile and can specify different kernel functions for the decision function. The GBDT model in this study utilized laboratory blood cell analysis data to predict MPP in children, achieving an impressive average AUC of up to 0.980. This suggests that, even at the early stage of MPP, the model can effectively identify affected individuals from the general population based solely on whole blood biomarkers. Additionally, the GBDT model provides feature importance ranking. When the top ten features are input as variables, the model demonstrates the best performance.

The pathogenesis of MPP remains incompletely understood, but it is commonly believed to involve two primary mechanisms: direct injury caused by MP and aberrant host immune responses [18]. MP infiltrates the respiratory tract, adheres to cell surfaces using adhesion organelles, and inflicts direct harm on respiratory epithelial cells by releasing oxygen free radicals and toxins [19]. Abnormal host immune responses to MP infection can result in immune-mediated damage to both pulmonary and extrapulmonary tissues via various pathways, including autoimmune reactions, allergic responses, and formation of immune complexes [20]. Basophilic granulocytes and monocytes, derived from bone marrow hematopoietic pluripotent stem cells, play pivotal roles in the inflammatory response associated with MPP, with basophils notably implicated in immune-related pneumonias [21]. Additionally, recent evidence has underscored the involvement of platelets beyond their traditional roles in thrombosis and hemostasis, particularly in inflammatory processes and infections [3, 22]. In this study, parameters such as absolute eosinophil value (Eos#), C-reactive protein (CRP), basophil ratio (baso%), platelet/lymphocyte ratio (PLR) and neutrophil count (Neut#) rank among the top ten in feature importance scores and serve as crucial predictors of MPP in children.

This indicates that the inflammatory response of the body may also be the main cause of MPP clinical symptoms [23, 24]. The change of cellular immunity plays an important role in the pathogenesis of MPP [25]. Basophils play an important role in immune diseases, and studies have also shown that basophils play a very important role in immune-associated pneumonia [26–28]. In this study, the proportion of basophilic granulocyte ranked first in the feature importance score, while the proportion of leukocyte and monocyte ranked third and tenth respectively. It is an important predictor of MPP in children. In recent years, more and more evidence has been found about the role of platelets outside thrombosis and hemostasis, such as inflammatory processes and infections. By combining thrombus and immune recruitment functions, platelets may contribute to hemostasis and immune response against potential infectious agents to prevent microbial invasion. PLR combines platelet count and lymphocyte count to better reflect the disease state. HCRP is a commonly used clinical indicator to reflect the infection of the body. It is a protein that increases acutely in the plasma when the body is infected or the tissue is damaged, with high sensitivity and low specificity. In this study, Eos#, CRP, Baso%, PLR, Neut#, PLT, Mono#, Mono%, HCT, and NLR ranked among the top ten in feature importance scores, which were also important predictors of MPP in children.

Exploring the pathogenesis of MPP is also an ongoing work. This study still has the following shortcomings: First, the included case information is retrospective data, and the results may be biased; Secondly, the examination items of children were different, and the data of some children were omitted due to missing indicators, and there may be omissions in predictive variables. Finally, the sample size was limited, no stratified study was conducted on children of different age levels, and the number of young children included was relatively small, and no prospective verification was conducted.

In this study, we developed a preliminary prediction model for pediatric MPP based on routine blood parameters using machine learning methods. Our findings underscore several key points worthy of discussion.Firstly, the successful development of a prediction model for pediatric MPP is significant due to the clinical challenges associated with its diagnosis. MPP presents with diverse clinical phenotypes, making accurate diagnosis challenging. Traditional diagnostic methods, such as clinical manifestations, radiological findings, and microbiological tests, lack specificity and may lead to misdiagnosis or delayed diagnosis. By leveraging machine learning techniques, we aimed to improve the accuracy and efficiency of MPP diagnosis, particularly in the pediatric population. Secondly, our study demonstrated the feasibility of using routine blood parameters for early identification of pediatric MPP infection. By analyzing 27 features extracted from routine blood tests, including white blood cell count, red blood cell count, and various differential counts, we constructed a classification model capable of distinguishing between MPP patients and non-MPP patients. Notably, our model achieved high AUC, accuracy, specificity, sensitivity, PPV, NPV and F1 score, indicating its potential clinical utility. Furthermore, our results highlight the importance of feature selection and model optimization in developing accurate prediction models. We employed seven commonly used machine learning algorithms and systematically evaluated their performance. Through hyperparameter tuning and model selection, we identified the GBDT algorithm as the best-performing model, achieving superior predictive performance compared to other algorithms. This underscores the significance of algorithm selection and parameter optimization in enhancing model performance. Additionally, the feature importance analysis revealed that Eos# and CRP were among the top contributing variables to the GBDT model. This suggests that alterations in platelet parameters may serve as potential biomarkers for pediatric MPP infection. Further investigation into the underlying mechanisms of platelet involvement in MPP pathogenesis may provide insights into disease pathophysiology and facilitate the development of novel diagnostic and therapeutic strategies.

Despite the promising results, our study has several limitations. Firstly, the retrospective nature of the study may introduce bias and limit generalizability. Prospective studies involving larger and more diverse patient cohorts are warranted to validate the performance of the prediction model in different clinical settings. Additionally, the lack of external validation in independent datasets necessitates further validation of the model’s robustness and generalizability.

## Conclusion

The machine learning algorithm based on blood routine parameters can provide clearer decision-making guidance for MPP diagnosis, effectively predict the occurrence and progression of MP infection, and provide a strong reference for clinical diagnosis and treatment, which is worthy of further clinical research and promotion.

### Electronic supplementary material

Below is the link to the electronic supplementary material.

Supplementary Material 1

## Data Availability

The dataset used and analyzed in this study is available from the corresponding author on reasonable request.

## Abbreviations

ML: Machine Learning
LR: Logistic Regression
KNN: K-Nearest Neighbors
NB: Naive Bayes
SVM: Support Vector Machine
DTM: Decision Tree
RF: Random Forest
XGBoost: Xtreme Gradient Boosting
GBDT: Gradient Boosting Decision Tree
ROC: Receiver Operating Characteristic Curve
AUC: Area Under the ROC Curve
PPV: Positive Predictive Value
NPV: Negative Predictive Value
PLT: Platelet Count
WBC: White Blood Cell Count
BASO: Basophils
EOS: Eosinophils
LYM: Lymphocytes
NEU: Neutrophils
RBC: Red Blood Cell Count
MCH: Mean Corpuscular Haemoglobin
MCHC: Mean Corpuscular Haemoglobin Concentration
MCV: Mean Corpuscular Volume
