# Supplementary material for: A preliminary prediction model of pediatric Mycoplasma pneumoniae pneumonia based on routine blood parameters by using machine learning method
Source: BMC Infect Dis. 2024 Jul 18;24:707. doi: 10.1186/s12879-024-09613-5 (PMC11264635; doi:10.1186/s12879-024-09613-5)
Supplement: Supplementary file 1 — Supplementary Material 1 [file 12879_2024_9613_MOESM1_ESM.docx]

**A preliminary prediction model of pediatric *Mycoplasma pneumoniae* pneumonia based on routine blood parameters by using machine learning method**

Xuelian Peng^1#^, Yulong Liu^1#^, Bo Zhang^1#^, Chunyan Yang^1^, Jian Dong^1^, Chen Yong^1*^, Baoru Han^2*^, Jin Li^1*^

*^a^ Department of Laboratory Medicine, The Affiliated Dazu’s Hospital of Chongqing Medical University, 402360, China*

*^b^ Medical Data Science Academy, College of Medical Informatics, Chongqing Medical University, Chongqing, 400016, China*

**Corresponding author:*

Jin Li, Email: [lijin@hospital.cqmu.edu.cn](mailto:lijin@hospital.cqmu.edu.cn);

Baoru Han, Email: [baoruhan@cqmu.edu.cn](mailto:baoruhan@cqmu.edu.cn);

Chen Yong, Email: [593489766@qq.com](mailto:593489766@qq.com).

Tel: +86-23-43780913

Fax: +86-23-43780913

#These authors contributed equally to this work

# SUPPLEMENTARY MATERIALS INDEX

**Figure S1.** **The overall** **flow chart of the predictive model Workflow diagram of our AI pediatric diagnosis framework.** This diagram depicts the process of constructing a dataset by collecting routine blood parameters and detecting MP RNA using RT-PCR. In the data preprocessing stage, the collected data undergoes preprocessing to prepare it for model training. Subsequently, multiple machine learning methods are utilized to construct the model, and the model with the best performance is selected.

**Figure S2. The training process of the model.** The training process of the model was divided into four stages: dataset construction, data processing, training and validation, and prediction.

**
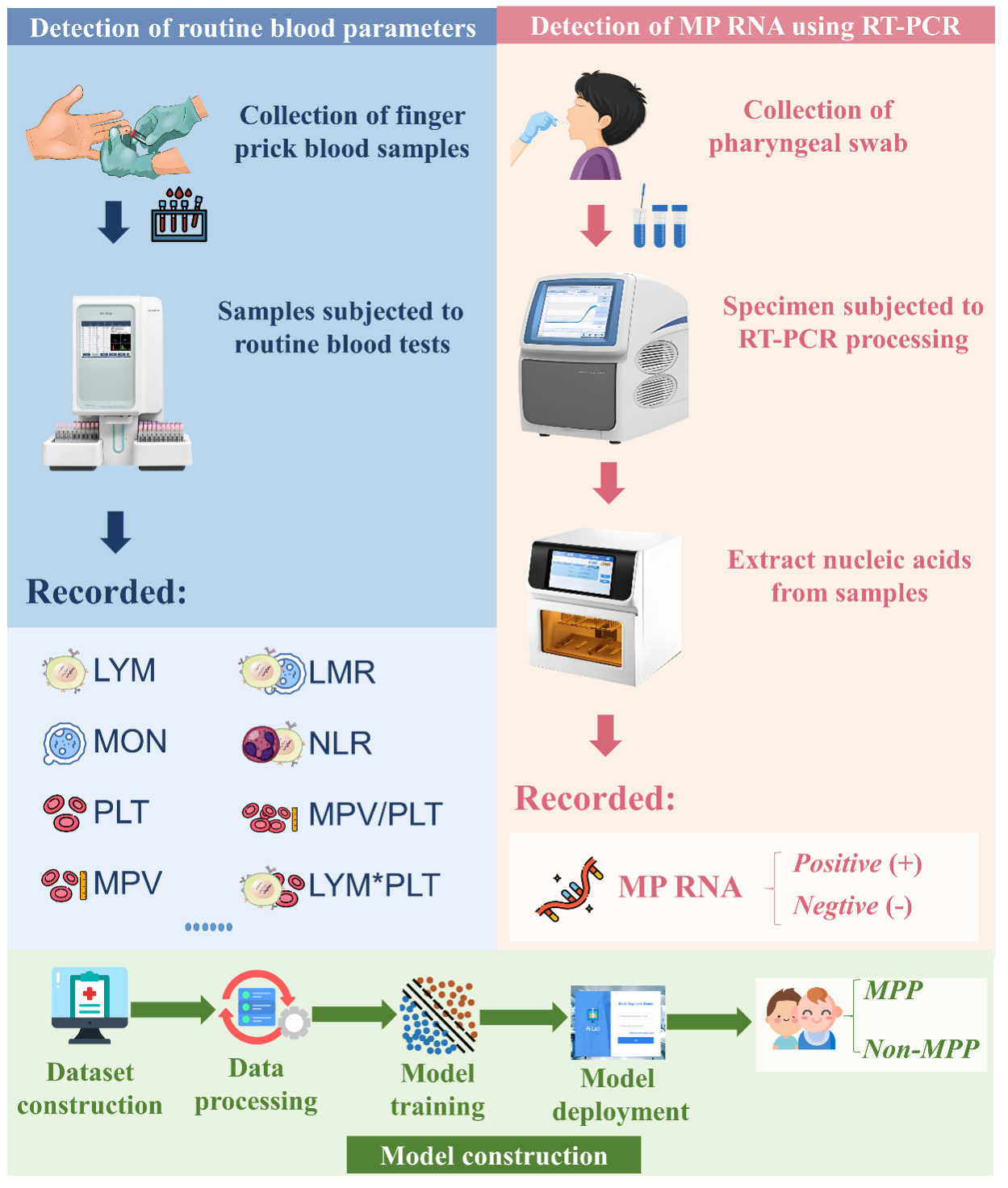
**

**Figure S1. The overall flow chart of the predictive model Workflow diagram of our AI pediatric diagnosis framework.**

**
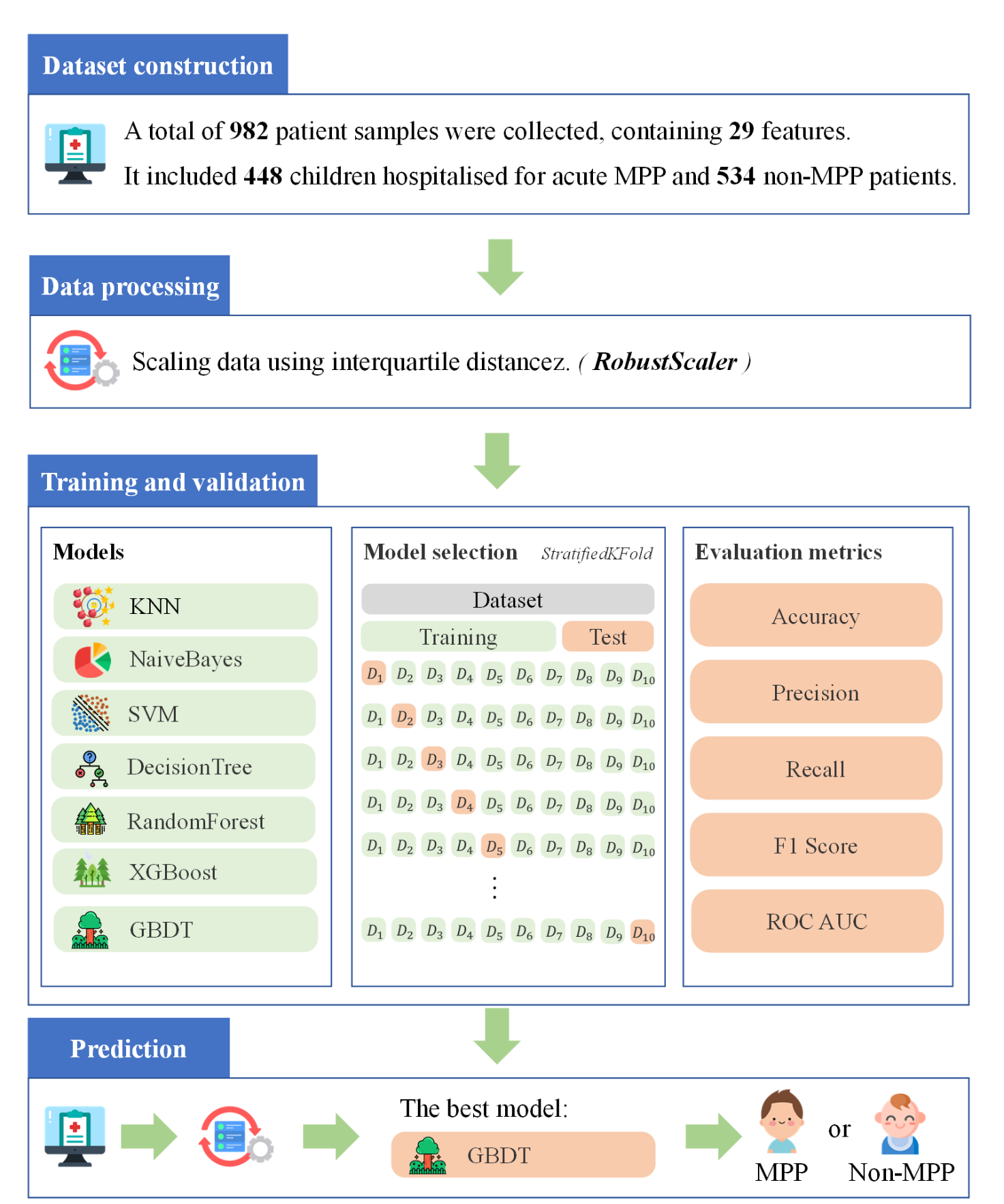
**

**Figure S2. The training process of the model.**
